# Cell-Penetrating Peptides Predicted From CASC3, AKIP1, and AHRR Proteins

**DOI:** 10.3389/fphar.2021.716226

**Published:** 2021-08-24

**Authors:** Ly Porosk, Kaisa Põhako, Piret Arukuusk, Ülo Langel

**Affiliations:** ^1^ Institute of Technology, University of Tartu, Tartu, Estonia; ^2^ Department of Biochemistry and Biophysics, Stockholm University, Stockholm, Sweden

**Keywords:** cell-penetrating peptides, AKIP1, CASC3, AHRR, prediction

## Abstract

Peptides can be used as research tools and for diagnostic or therapeutic applications. Peptides, alongside small molecules and antibodies, are used and are gaining further interest as protein-protein interaction (PPI) modulators. Peptides have high target specificity and high affinity, but, unlike small molecule modulators, they are not able to cross the cell membranes to reach their intracellular targets. To overcome this limitation, the special property of the cell-penetrating peptides (CPPs) could benefit their cause. CPPs are a class of peptides that can enter the cells and with them also deliver the attached cargoes. Today, with the advancement of in silico prediction tools and the availability of protein databases, designing new and multifunctional peptides that are able to reach intracellular targets and inhibit certain cellular processes in a very specific manner is reachable. Although there are several efficient CPP sequences already known, the discovery of new CPPs is crucial for the development of efficient delivery methods for both biotechnological and therapeutic applications. In this work, we chose 10 human nuclear proteins from which we predicted new potential CPP sequences by using three different CPP predictors: cell-penetrating peptide prediction tool, CellPPD, and SkipCPP-Pred. From each protein, one predicted CPP sequence was synthesized and its internalization into cells was assessed. Out of the tested sequences, three peptides displayed features characteristic to CPPs. These peptides and also the predicted peptide sequences could be used to design and modify new CPPs. In this work, we show that we can use protein sequences as input for generating new peptides with cell internalization properties. Three new CPPs, AHRR_8-24_, CASC3_251-264_, and AKIP1_27-37_, can be further used for the delivery of other cargoes or designed into multifunctional peptides with capability of internalizing cells.

## Introduction

There are many macromolecules with high potential in therapeutic and biotechnological applications. Nonetheless, their use is limited due to their inability to efficiently cross cell membranes to reach intracellular targets. Successful modulation of various cellular processes would broaden the possibilities in both fields. For the delivery of macromolecules, different physical, biological, and chemical delivery methods have been developed. One of the chemical methods is the use of CPPs. CPPs are generally defined as 4–40 amino acid (aa) long peptides that can enter cells and with them enhance the cellular uptake of associated cargo. The first reporting of protein transduction was already over 3 decades ago, when two independent groups described the cellular uptake of Tat protein of HIV-1 (Frankel and Pabo, 1988; Green and Loewenstein, 1988). Over the years, many more CPP sequences have been successfully discovered and designed. There are over 1,700 sequences of experimentally validated CPPs (Agrawal et al., 2016). The most common CPP is a linear, synthetic peptide containing L-aa. The majority of validated CPPs are either synthetic or protein derived (Supplementary Figure S1A). Attachment of fluorophore to the CPP sequence is often used to confirm the cell penetration ability (Supplementary Figure S1B), although different cargoes, such as nucleic acids, proteins, and PNAs, are possible. For further modification, peptide sequences or other cargo can be attached to mediate other bio functions, such as PPI modulation and cell proliferation.

The main advantages of the peptides and CPPs are their ease of synthesis and possibility to include variety of modifications; and as they are biocompatible, they are generally well tolerated by the cells. In addition to these, due to the advancement of in silico prediction approaches and the availability of different databases, designing new and unique CPPs with the possibility to affect cells in a specific manner is tangible. Protein databases, with over 200,000 entries, can be used as sources for predicting new CPPs. Different parameters, like protein cellular location, association with diseases, known structure, and so forth, could be used to refine the choice of input. Protein sequences often include specific motifs, which encode functions like their intracellular trafficking or their interactions with other nucleic acids or other proteins. Nuclear trafficking sequences, such as nuclear localization sequences (NLS) or nuclear export signals (NES), are found in several proteins. They direct the protein transport or shuffling between the cytoplasm and nucleus. The NLS motifs, with some exceptions, are characterized by one or more clusters of positively charged residues with the consensus sequence of K-K/R-X-K/R (Xiong and Blainey, 2016). These are preceded by helix-breaking residue or separated by several residues. Molecular sleds, which have been shown to slide along DNA (Zhang et al., 2017a) and may influence interactions with it, consist of spans of arginines or lysines or combinations of these. The inclusion of NLS sequences in the CPP would be beneficial if the peptide and its associated cargo must reach the nucleus. Additional molecular sled motif would benefit when the interaction with the nucleic acid is desired.

In this work, we aimed to find new CPPs from human proteins by using different prediction programs followed by the synthesis and assessment of internalization of these peptides.

## Materials and Methods

### Prediction of Cell-Penetrating Peptides

For the CPP predictions in this work, 10 FASTA format reviewed human nuclear protein sequences were selected from UniProtKB database (https://www.uniprot.org/) and screened for potential CPP sequences.

For the first round of predictions, only one prediction program was used, based on the works of Hällbrink et al., 2005, and Hansen et al., 2008. This predictor uses Z-scales for each aa based on their physicochemical descriptors, followed by subjection to partial least squares (PLS) and principal component analysis (PCA). For training, 85 non-CPP and CPP sequences were used. The advantage of this predictor is that the whole protein sequence can be inserted for prediction, whereas most of other predictors have limited input sequence lengths. This helps to identify specific regions in the protein where most probable CPP sequences could be located. The protein sequence was fractioned into peptides with the length from 5 to 30 aa and screened. The output of the predictor is a .txt file with scored sequences. The data was refined with Notepad++ by extracting only the sequences that were scored “2” or “3,” as they are more likely to have CPP properties. These sequences were used as an input for other predictors and for further selection for peptide synthesis. Both NLS and sled motifs were screened from these predicted CPP sequences. Motifs included were K-K/R-X-K/R, where X is any amino acid or span of amino acids, known NLS regions (UniProt database), and YYYY or YYY, where Y is K or R in any combination.

CellPPD (Gautam et al., 2013) takes into account the aa composition, physicochemical properties, pattern profiles, and motifs, and it uses machine learning algorithm that is based on the support vector machine (SVM) method. For training, a much larger dataset of 708 CPPs was used. It was reported that the prediction accuracy is increased compared to the previous predictors. The “Multiple Peptides” was used for SVM based screening as well as SVM + motif-based method, with e-value cut-off set at 10 and SVM threshold at 0.0. The output is a table with prediction and score and, if chosen, the physicochemical properties were also calculated.

SkipCPP-Pred is a predictor developed by (Wei et al., 2017). It is a further development of the CPP predictors. It is a two-layer predictor, which uses features of dipeptides instead of single residues. Four feature descriptors were used and then ranked using the minimum redundancy maximum relevance (mRMR) algorithm. The optimal feature subset was selected and used to train random forest (RF). In the SkipCPP-Pred, compared to CPPred-RF, the peptide sequence is processed by a k-skip-n-gram model. The predictor does not allow setting any other values and only sequence will be used as input. The output is a prediction (CPP or non-CPP) and the confidence of the prediction (0–1).

All sequences that were predicted as non-CPP or had a very low score in SkipCPP-Pred (score below 0.7) were excluded. Out of these, 10 sequences from 10 proteins were chosen for synthesis.

### Synthesis of Peptides

The peptides were synthesized on an automated peptide synthesizer (Biotage Initiator+ Alstra) using the fluorenylmethyloxycarbonyl (Fmoc) solid phase peptide synthesis strategy with Rink-Amide ChemMatrix resin (0.45 mmol g^−1^ loading) to obtain C-terminally amidated peptides. For fluorescently labelled peptides, the fluorescent label (FAM) 5(6)-Carboxyfluorescein was coupled manually to the N-terminus of the peptide overnight at room temperature with 5 eq. The reaction was carried out using HOBT/HBTU as coupling reagents in DMF, with DIEA as an activator base.

Cleavage was performed with trifluoroacetic acid, 2.5% triisopropylsilane, and 2.5% water for 2 h at room temperature. The peptides were purified by reversed-phase high-performance liquid chromatography on a C4 column (Phenomenex Jupiter C4, 5 μm, 300 Å, 250 × 10 mm) using a gradient of acetonitrile/water containing 0.1% TFA. The molecular weight of the peptides was analyzed by matrix-assisted laser desorption-ionization/time of flight mass spectrometry (Bruker microflex LT/SH, United States). The concentration of the peptides was determined based on the dilutions of accurately weighed substances.

### Cell Experiments

U87 (human glioblastoma like cells) and HeLa (human cervical cancer) were maintained at 37°C and 5% CO_2_ in Dulbecco’s Modified Eagle’s Medium (DMEM) (Sigma, Germany). PC3 (human prostate cancer) and CHO cells were maintained in Ham´S F12 media and incubated at 37°C and 5% CO_2_. Cell media were supplemented with 0.1 mM non-essential amino acids, 1.0 mM sodium pyruvate, 10% fetal bovine serum (FBS) (Sigma, Germany), 100°U ml^−1^ penicillin, and 100 mg ml^−1^ streptomycin (Invitrogen, Sweden). U87 cell dishes were treated with 0.1% gelatin solution prior to addition of cells and media.

#### Internalization of the Fluorescently Labelled Peptides

For assessing the internalization of the peptides from the cell lysate, 50,000 cells were seeded on a 24-well plate 1 day prior to the experiment. On the experiment day, cell media was changed to 450 µl of fresh serum free media followed by the addition of 50 µl of fluorescently labelled peptide solution. The peptide’s final concentration on the cells was 10 µM. The cells were incubated with the peptides for 2 h, following washes with PBS buffer and cell lysis with 100 µl of 0.1% Triton X100. The cell lysate was transferred to black 96-well plate and the fluorescence signal from the fluorescently labelled peptide was measured.

Total DNA was measured from U87 cells 48 h after the addition of the peptide solutions. After the incubation, the cells were washed with PBS and lysed as described above. From the cell lysate, DNA was quantified with Quant-iT™ PicoGreen™ dsDNA Assay Kit. The results are shown as the average of measurements from three wells and normalized to untreated cells.

The percentage of the fluorescent cells after peptide treatment was assessed using flow cytometry. For flow cytometry, 10,000 cells were seeded on a 96-well plate 1 day prior to the experiment. On the experiment day, the cell media was changed to 90 µl of fresh serum free media followed by the addition of 10 µl of the fluorescently labelled peptide solution. The peptide’s final concentration on the cells was 10 µM. The cells were incubated with the peptides for 2 h, following washes with PBS buffer. 0.25% Trypsin-EDTA was added to detach the cells from the plate and 200 µl of PBS mixed with 1% FBS was added. Shortly before the analysis, 50 µl of 0.4% trypan blue was added to quench the fluorescence outside the cells. The fluorescence detected from the untreated cells was used to set the signal threshold. The results are shown as the percentage of the fluorescent positive cells.

48 h prior to the addition of the cells, 20,000 HeLa cells were seeded on 8-well Nunc™ Lab-Tek™ (Thermo Scientific™, United States) chamber plates. Before the addition of the peptide solutions media was changed to 225 µl of fresh media. The final concentration of the peptide on the cells was 10 µM. After 4 h incubation, the cells were washed with PBS and counterstained with Hoechst 33342. Confocal images were acquired from live cells with Zeiss LSM710 (Carl Zeiss AG, Germany). For detecting the fluorescent label from the peptide, 488 nm laser with 493–490 filter was used, and for detecting nuclei 405 nm laser with 416–503 filter was used. The images were taken with 20x magnification.

#### Toxicity Assay (MTS)

The cell proliferation was analyzed with the CellTiter 96® Aqueous Non-Radioactive Cell Proliferation Assay (MTS) (Promega Biotech AB, Sweden) according to the manufacturer’s instructions. For this, 10,000 U87 or CHO cells were seeded 1 day prior to the experiment on transparent 96-well plates. On the experiment day, media was replaced with 90 μl of fresh serum free media. Peptides in the concentration range from 0 to 32 µM were added to the cells. The cells were incubated with the peptides for 20 h or 46 h, following the addition of the reagent and further incubation to reach 24 h or 48 h timepoints. The absorbance of the formazan product was measured at 490 nm with Tecan Sunrise microplate absorbance reader (Tecan Group Ltd., Switzerland) and the percentage of viable cells was calculated using the GraphPad Prism software 5.0 (GraphPad Software, CA, United States).

#### Statistical Analysis

Two-way ANOVA was used for the statistical analysis of the treatment groups. ns indicated *p* > 0.05; * indicated *p* < 0.05; ** indicated *p* < 0.01; *** indicated *p* < 0.001. The groups that were compared are indicated in the figure captions.

### Results

#### Prediction of CPPs From the Protein Sequences

For this work, the amino acid sequences of one isoform from 10 human nuclear proteins were selected (Table 1 and Supplementary Table S1) and screened with the CPP predictor (Hällbrink et al., 2005; Hansen et al., 2008). The first round of predictions allowed screening of the whole protein which was made into peptides of a length of 5–30 aa and then individually predicted for the probability of being a CPP. We limited the length of the predicted CPPs to 30 aa, as other prediction programs have size limitations for the sequences. Additionally shorter peptides would be preferred as the CPP if cargoes, such as bioactive peptides, will be further added. From each protein, all the predicted CPP sequences that scored higher than 1 (scale from 1 to 3, where 2 or 3 are more probable of being a CPP, number of suitable sequences shown in Table 2, and scored sequences in Supplementary Table S3) were further screened with the online predictors CellPPD and SkipCPP-Pred (Gautam et al., 2013; Wei et al., 2017; Porosk et al., 2020). From each protein, one potential CPP sequence was chosen for the synthesis and characterization (Table 2 and Supplementary Table S2). As a control, a known CPP, Tat_48-60_, was included. The prediction of new CPPs is becoming a more relevant tool for pre-screening of protein sequences, with a recent example of SARS-COV-2 proteome screening (Kardani and Bolhassani, 2021).

**TABLE 1:** Chosen proteins and parameters (data from UniProtPK or The Human Protein Atlas).

	Database					
	UniProtPK			The Human Protein Atlas		
Protein/encoding gene, aliases	Isoforms	Length (aa)^a^	Structure available (yes/no)	Data available	Location	Cancer prognostic or protein expression in malignant tissues
AKIP1 (BCA3, C11orf17)	5	210	N	RNA	Intracellular, nucleoplasm	Prognostic marker for liver and endometrial cancer
CASC3 (BTZ, MLN51)	3	703	Y	RNA/protein	Intracellular, nuclear membrane	Gliomas, testicular, urothelial, stomach, colorectal and liver cancers: moderate
CCNL2 (ania-6b, CCNM, CCNS, HLA-ISO, PCEE, SB138)	5	520	N	RNA/protein	Intracellular, nucleoplasm	Prognostic marker in urothelial and renal cancer
DAPK1 (DAPK, ROCO3)	4	1,430	Y	RNA/protein	Membrane, centrosome	Prognostic marker for colorectal cancer
ING4 (my036, p29ING4)	8	249	Y	RNA/protein	Intracellular, nucleoplasm	Prognostic marker for lung cancer
DMAP1 (DNMAP1, DNMTAP1, EAF2, FLJ11543, KIAA1425, MEAF2, SWC4)	6	467	Y	RNA/protein	Intracellular, nucleoplasm, and cytosol	Prognostic marker for liver cancer. Moderate staining in breast, thyroid, and testicular cancers
NOP53 (GLTSCR2, PICT-1, PICT1)	5	478	N	RNA/protein	Intracellular, nucleoli	Prognostic marker for endometrial cancer. Weak-to-moderate staining in most malignant cells
AHRR	3	701	Y	RNA	Intracellular, nucleoplasm, and cytosol	Low cancer specificity
CUL4B	3	913	Y	RNA/protein	Intracellular, nucleoplasm	Moderate staining in few cases of most cancers
FBXO32	2	355	N	RNA	Intracellular, nucleoplasm, and cytosol	Prognostic marker in renal cancer

a Length of the isoform shown in ST2, if not marked differently. The longest available sequence is chosen.

**TABLE 2:** Predicted CPP sequence, calculated molecular weight, and prediction scores for peptides.

Predicted CPP sequence^a^	Peptide name	Mw^b^	Prediction scores		
			Possible CPPs from protein^c^	CellPPD^d^	SkipCPP-Pred^e^
GRKKRRQRRRPPQ	Tat_48-60_	1719.21	—	1.37/CPP	0.98
VLERAKRRAV	AKIP1_27-37_	1997.58	322	0.14/CPP	0.94
PDDIKPRRIRKPRY	CASC3_251-264_	1810.32	86	0.24/CPP	0.79
NTKRRLEGAKKA	CCNL2_354-365_	1371.77	319	0.11/CPP	0.93
AAKFIKKRRTKSS	DAPK1_40-52_	1521.01	152	0.13/CPP	0.95
TQKEKKAARARSK	ING4_134-145_	1501.92	476	0.03/CPP	0.9
RKRRESASSSSSVKKAKKP	DMAP1_459-467_	2117.68	348	0.31/CPP	0.92
KRKGRLRSKGKK	VP8	1441.96	—	0.63/CPP	1
AEADKPRRLGRLK	NOP53_397-410_	1509.95	1,178	0.06/CPP	0.93
GECTYAGRKRRRPLQK	AHRR_8-24_	1919.46	197	0.31/CPP	0.87
TPPTSAKKRKL	CUL4B_48-59_	1226.63	309	0.34/CPP	0.97
VAAKKRKKDML	FBXO32_59-69_	1287.78	47	0.26/CPP	0.73

a All synthesized peptides are amidated at C-terminus.

b Molecular weight calculated with CellPPD.

c Number of predicted CPP sequences between 5 and 30 aa, shown only number of sequences that scored 3. Scale 0–3, where 3 is most likely to have CPP properties. The program is based on the works of Hällbrink et al., 2005, and Hansen et al., 2008.

d SVM score and based on prediction it is a non-CPP or a CPP. It must be noted that CPP Tat is included in the CellPPD training set, explaining the score above 1, although the range is 0–1 (Gautam et al., 2013).

e Score between 0 and 1, where 1 is most likely to be a CPP (Wei et al., 2017).

The 10 chosen proteins have distinct functions in the cells, although their specific functions were not the basis of selection. A-kinase-interacting protein 1 (AKIP1) is a protein located in the cell nucleoplasm (Sastri et al., 2005), and its known function is related to enhancement of the NF-kappa-B transcriptional activity. It regulates the nuclear localization of the NF-kappa-B subunit RELA and promotes the phosphorylation of RELA by PRKACA. Additionally, it regulates the effect of the cAMP-dependent protein kinase signaling pathway on the NF-kappa-B activation cascade (Gao et al., 2008; Gao et al., 2010). It is also related to the liver and endometrial cancer (Zhang et al., 2018; Fang and Lu, 2020) and their prognosis. The metastatic lymph node 51 (CASC3, MLN51) protein is a required component of the spliceosome (Zhang et al., 2017b) which is responsible for pre-mRNA splicing in the cells. It is located mainly in the nuclear speckles (Ballut et al., 2005; Zhang et al., 2017b). The nuclear speckles or the splicing speckles are where the splicing factor storage and modification in the nucleus take place. The exon junction complex (EJC) is a multiprotein complex, where CASC3, through interacting with eIF3, activates translation (Chazal et al., 2013). It is also related to the stress response in the cells, promoting its recovery following stress (Baguet et al., 2007). The Aryl hydrocarbon receptor repressor (AHRR) protein is found in the cell nucleoplasm and cytoplasm. AHRR is enriched in the testis tissues. It is a regulator of transcription (Haarmann-Stemmann et al., 2007) and is involved in the regulation of the cell growth and differentiation. It represses the transcription activity of the aryl hydrocarbon receptor. AHRR has been considered a regulator in the cancer cells and a possible target for treatment (Zudaire et al., 2008).

Cyclin-L2 (CCNL2) is a protein belonging in the cyclin family, which controls the passage of the cell cycle. CCNL2 functions as a regulator for pre-mRNA splicing. It interacts with several proteins, including RNA polymerase II, cyclin-dependent kinases, and splicing factors. It is in the nucleoplasm and the nuclear speckles of the cell. Additionally, it may induce apoptosis through the modulation of the apoptotic/antiapoptotic protein expression (Yang et al., 2004; Loyer et al., 2008). Death-associated protein kinase 1 (DAPK1) is a calcium/calmodulin-dependent serine/threonine kinase involved in several cellular signaling pathways, which are responsible for the cell survival, apoptosis, autophagy (Singh et al., 2016), and suppression of the necroptosis (Wu et al., 2020). The protein is located in the cytoplasm. Inhibitor of growth protein 4 (ING4) is a member of ING family, and it is located in the cell nucleus. It is involved in several cellular processes as both positive and negative regulator. It has been shown to be involved in the inhibition of the cell growth (Soliman and Riabowol, 2007) and is possibly a regulator of the rRNA synthesis (Trinh et al., 2019). It is known to interact with p53 and NF-κB (Jafarnejad and Li, 2012; Hou et al., 2014), affecting the cell proliferation. DNA methyltransferase 1-associated protein 1 (DMAP1) is located in both the cell cytoplasm and the nucleus. It is involved in several cellular functions as a transcription repressor and activator (Xin et al., 2004; Lee et al., 2010). Ribosome biogenesis protein NOP53 (NOP53) is located in the cell nucleus and is involved in the regulation of several cell functions, including integration of 5S RNP into ribosomal large subunit, ribosome biogenesis, sensor regulating the activation of p53/TP53 in response to stress conditions, and tumor suppression (Sloan et al., 2013; Chen et al., 2016a; Chen et al., 2016b). Cullin-4B (CUL4B) is a core component of the cullin-RING-based E3 ubiquitin-protein ligase complex. The complex mediates the ubiquitination and subsequent proteasomal degradation of the target proteins. Among other functions, CUL4B regulates the mTOR pathway which is involved in the control of the cell growth, the cell size, and the metabolism (Higa et al., 2006; Zou et al., 2009; Badertscher et al., 2015). F-box only protein 32 (FBX32) is a substrate recognition component of a SCF (SKP1-CUL1-F-box protein) E3 ubiquitin-protein ligase complex which mediates the ubiquitination and the subsequent proteasomal degradation of target protein (Tintignac et al., 2005). It is located in both the cytosol and the nucleus (Julie et al., 2012; Al-Hassnan et al., 2016).

The prediction was based on the prediction program (Hällbrink et al., 2005; Hansen et al., 2008). In AKIP1, most of the predicted CPPs were in the N-terminal part of the protein sequence, more specifically in the first quarter of the 210 aa protein. In CASC3, there are three potential regions predicted with the highest number of possible CPPs (roughly aa 50–70, 200–250, and 400–450). In AHRR, two regions had the most predicted CPP sequences, N-terminal region aa 20–50 and middle region aa 280–320. In CCNL2, two regions from aa 140–190 and 330–390 had the highest number of predicted CPPs. In DAPK1, the region aa 30–65 included mainly the predicted CPPs. In ING4, there were three regions aa 120–170 (includes bipartite NLS), 270–310, and 650–700 that included most of the predicted CPP sequences. In DMAP1, only one distinct region included most of the predicted CPPs sequences, aa 250–320. In NOP53, three regions aa 80–120, 330–370, and 400–424 included the most predicted CPP sequences. In CUL4B, there were five regions with predicted CPP sequences: aa 190–210, 550–580, 600–620, 770–800, and 850–870. In FBX32, aa 270–310 included most of the predicted CPP sequences. The regions are marked in Supplementary Table S1.

In the second round, we used as an input the sequences from the first round which scored 2 and 3 (sequences shown in Supplementary Table S3). CellPPD and SkipCPP-Pred (Gautam et al., 2013; Wei et al., 2017) were used for this. Interestingly, for batch analysis, the peptide sequences from AHRR were predicted as non-CPP with CellPPD, but when predicting individually, most of the peptides were predicted as CPPs (Supplementary prediction data). The CellPPD size limit for prediction was 10 aa; therefore, shorter peptides were excluded. All the CPP sequences predicted from the first round were also predicted to be CPP with high probability (Supplementary Table S3).

The main advantage of the first prediction program is that the whole protein sequences can be used as the input, and specific regions, which have high probability of being CPPs, can be mapped in the protein. These regions, when they co-locate with protein PPI sites or other functional sites, could be used to design multifunctional peptides or be used for creating fusion peptides between predicted CPP sequences and peptides with known functions, both from the same protein. Most of the peptides that scored 3 in the first prediction round had a high probability score also with CellPPD and SkipCPP-Pred, which may encourage limiting the predictions to only the first prediction round. The advantage of CellPPD is that it also calculates other parameters for the peptide sequence and the prediction parameters can be further defined. The additional advantage is the possibility to introduce one amino acid changes to the sequence to predict if this could result in a better score. The input data is also easy to handle. In SkipCPP-Pred, the peptide sequences must be converted in a specific format, which requires additional steps before the predictions. All three have their own limitations and advantages, discussed in the work of (Porosk et al., 2020).

#### Internalization of the Fluorescently Labelled Peptides Into the Cells

**FIGURE 1:**
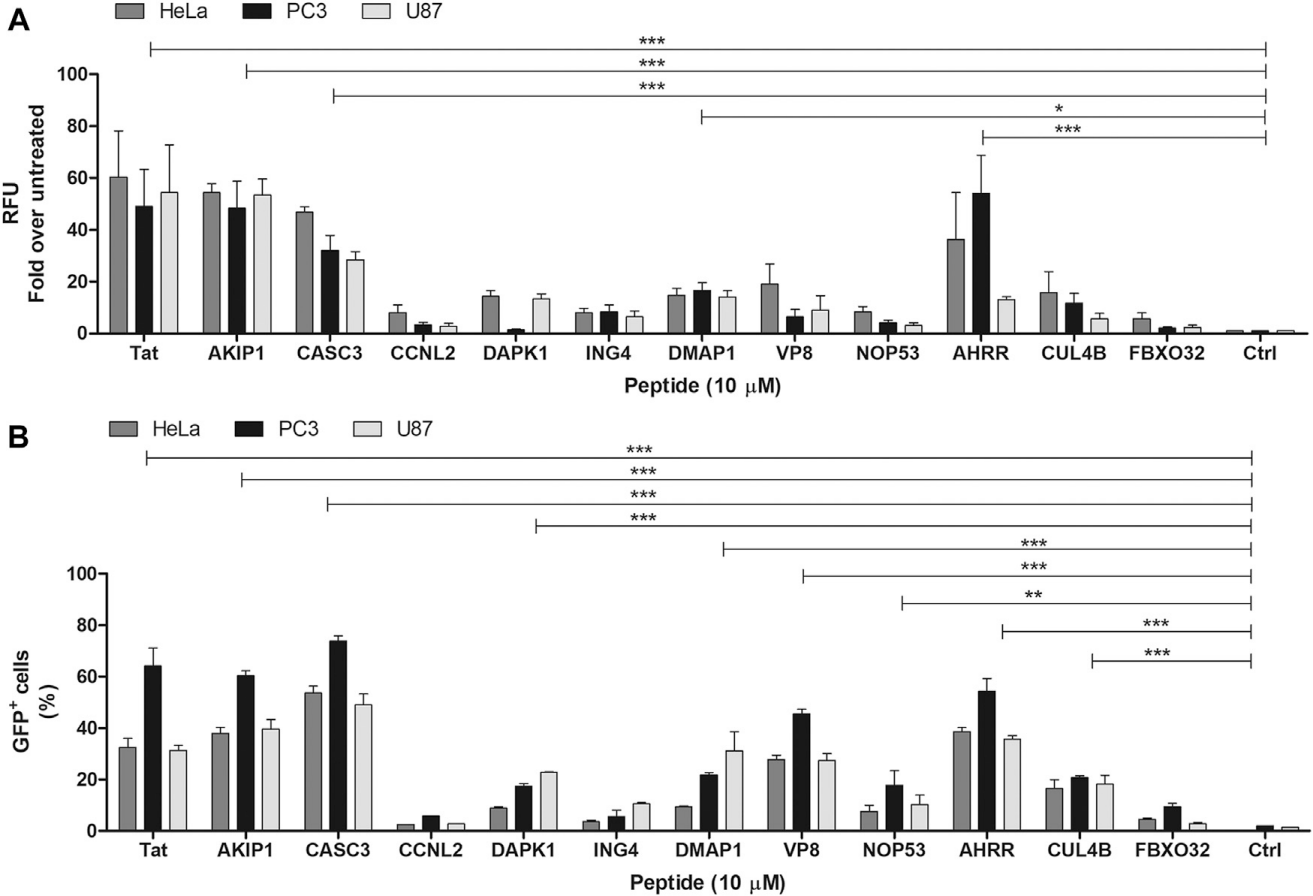
**(A)** Internalization of the FAM-labelled peptides into the U87, PC3, and HeLa cells. The peptide’s concentration on the cells was 10 µM. The fluorescence signal measured from the cell lysate 2 h after the addition of the peptide to the cells. The results are expressed as fold over untreated cells. The peptides names on the graph are shown as the protein names they are derived from (e.g., without location in the protein sequence). **(B)** Percentage of the labelled cells after the addition of the fluorescently labelled peptides. Measured from the U87, PC3, and HeLa cells. The peptide’s concentration on the cells was 10 µM. The fluorescent signal detected 2 h after the addition of the peptides to the cells. The untreated cells were used to set the threshold for the fluorescent signal. Two-way ANOVA with Bonferroni post-test was used for the statistical analysis of the treatment groups to untreated control. ns (not marked) indicates *p* > 0.05; * indicates *p* < 0.05; ** indicates *p* < 0.01; *** indicates *p* < 0.001.

**FIGURE 2:**
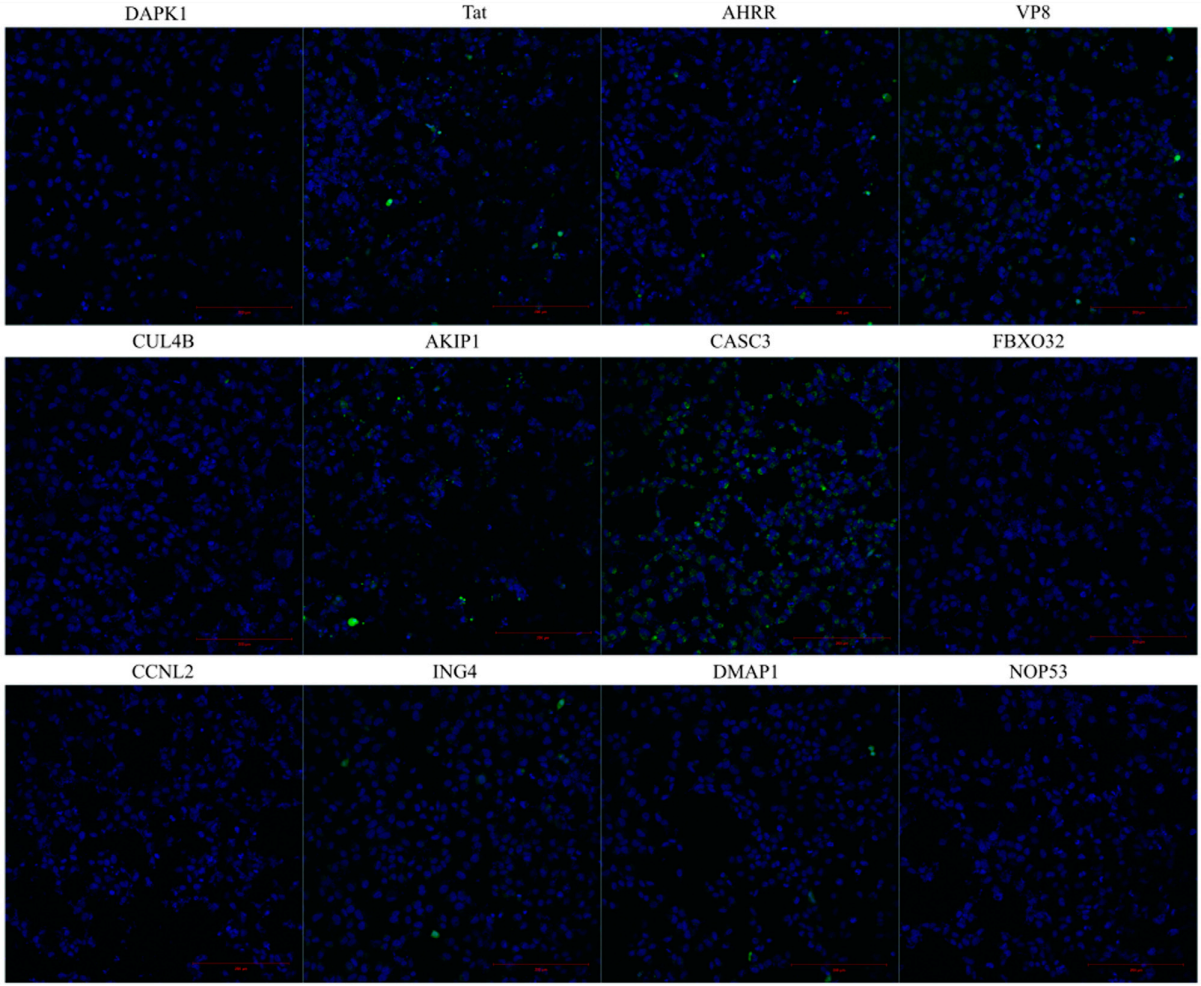
Internalization of the fluorescently labelled peptide into the HeLa cells. The images are taken from live cells 4 h after the addition of the fluorescently labelled peptide to the cells. The peptide’s final concentration on the cells was 10 µM. Hoechst was used to stain the nuclei.

CPPs are peptides that can enter the cells. We tested the internalization of the predicted and synthesized peptides by measuring the fluorescence signal from the cell lysates after the treatment with the fluorescently labelled peptides. It should be noted that measuring signal from lysate is unable to distinguish the internalization between the labelled peptide and the peptides attached on the membrane. Out of all the tested peptides, AKIP1_27-37_, CASC3_251-264_, and AHRR_8-24_ showed the highest fluorescence intensities, although all the peptides had higher fluorescence than the untreated background (Figure 1A), albeit only 1-2-fold. The internalization of the peptides was confirmed also with flow cytometry and with confocal microscopy (Figures 1B, 2 respectively). Interestingly, in addition to AKIP1_27-37_, CASC3_251-264_, and AHRR_8-24_, other peptides showed fluorescent signal in more than 10% of the cells. The differences between the measurement from the cell lysate and the flow cytometry may be explained with differences in the sensitivity of detection. Nevertheless, in both the cell lysate and flow cytometry, mainly the same peptides AKIP1_27-37_, CASC3_251-264_, and AHRR_8-24_ were highlighted. In the cell lysate experiment, the signal from DMAP1 was also considered as above control (Figure 1A). In flow cytometry (Figure 1B), in addition to what was previously mentioned, all the peptides except the CCNL2, ING4, and FBXO32 derived peptides had a detectable signal. In the confocal microscopy, the incubation time was increased from 2 to 4 h, as at 2 h the signal was hardly distinguishable from the background.

**FIGURE 3:**
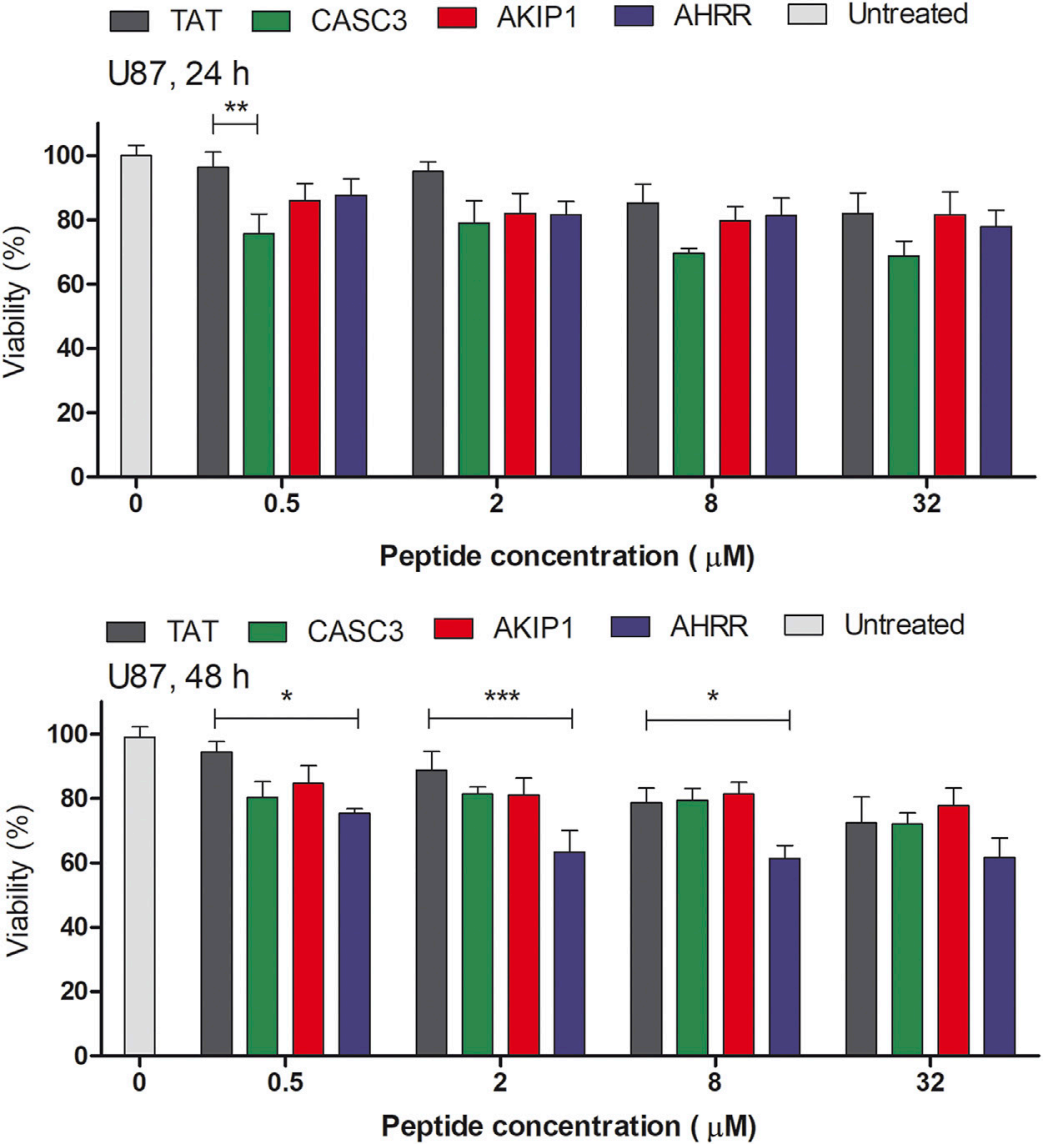
MTS assay on the U87 cells in serum free media. The absorbance was measured at 24 and 48 h time points after the addition of the peptides to the cells. The tested peptides’ range was between 0 and 32 µM. The results from the absorbance measurement are normalized to the untreated cells (100%). Two-way ANOVA with Bonferroni post-test was used for the statistical analysis of the treatment groups to control CPP Tat treated group at the same concentration. ns (not marked) indicates *p* > 0.05; * indicates *p* < 0.05; ** indicates *p* < 0.01; *** indicates *p* < 0.001.

When comparing the treated cell population, the percentages between U87 and HeLa cells did not significantly differ in the total fluorescence measured from the cell lysates, whereas the percentages were higher in PC3 cells (statistical analysis results in Supplementary Table S4). In the cell lysate analysis, there were no statically significant differences between the cell lines. In the confocal images (Figure 3), the fluorescence signal from the peptides can be seen in the cells treated with AKIP1_27-37_, CASC3_251-264_, and AHRR_8-24_ and CPP Tat. Interestingly, at given experimental conditions, the strongest and well dispersed signal was detected with CASC3_251-264_ (Figure 2). We additionally tested 20 µM concentrations for CASC3_251-264_ (Supplementary Figure S2) in HeLa cells and compared it to CPP Tat at the same concentration and in the same experimental setup as described for 10 µM confocal images. At 20 µM concentration, both peptides were able to enter into almost all the cells, although the intensities varied.

#### Effect of the Peptides on the Cell Proliferation

The amount of DNA may reflect the proliferation of cells (Supplementary Figure S3). At 5 µM concentration, AKIP1_27-37_ treated group had a decreased amount of total DNA after 48 h. In U87 cells and after 24 or 48 h, the CPP Tat treated cells did not show a considerable amount of toxicity (Figure 3 and Supplementary Figure S4, statistical analysis results in Supplementary Table S4). Interestingly, the CASC3_251-264_ treated cell proliferation decreased at higher 8 and 32 µM concentrations in both the CHO and U87 cells. In the U87 cells and after 48 h, the AHRR_8-24_ treated cells showed decreased viability. The CHO cells are affected less by the peptides. Although the U87 cells show a decrease in the proliferation after the peptide treatment, the cells themselves are more sensitive to different manipulations, compared to, for example, the CHO cells. We chose the CHO and U87 cells as representatives of the widely used cell line (CHO) and sensitive cell line (U87-MG).

When compared to the CPP Tat treated group instead of the untreated group, there was no statistically significant difference between other peptide treated groups at the same concentration, except for 0.5 µM CASC3_251-264_ at 24 h and 0.5, 2, and 8 µM AHRR_8-24_ at 48 h in the U87 cells (Figure 3), where a decrease in the proliferation was observed.

The mechanism behind the decrease of the total DNA amount and the proliferation assessed by MTS might be related to the delayed damage to the cell membranes, although the peptides are considered relatively safe. It should be noted that the MTS assay is carried out in serum free media, as the addition of serum may decrease the effect of the peptide on the cells.

## Discussion

There are many diseases where the modulation of PPI could alleviate the disease state. Additionally, it has a broad applicability in research to investigate different cellular processes. Although today there are several prediction programs available, with their own limitations and advantages (Porosk et al., 2020), there is a strong predicament to test the predicted sequences. Still, with so many prediction tools and databases available, designing proteins and peptides into CPPs is easier than ever before. The availability of large databases has made the discovery and characterization of new CPPs and, more importantly, the design of multifunctional peptides feasible. For example, many CPPs have been predicted and characterized from SARS-COV-2 using bioinformatics approaches (Kardani and Bolhassani, 2021).

The key in the current state of the CPP field is including more than one function into one peptide. This would open new opportunities for designing efficient multifunctional peptides, for example, CPPs that are PPI inhibitors. In this work, we show, based on 10 chosen proteins and their sequences, that, by using CPP prediction tools, we can successfully find new CPPs. The predicted CPP sequences from each protein of interest can be chosen based on different aspects, for example, to include specific motifs and include specific regions from the protein. The availability of abounding modification possibilities, such as stapling and inclusion of non-coded amino acids, offers immense prospects for using peptides as biomimetic or PPI modulators.

The peptides derived from all three proteins could be potential PPI regulators to alleviate disease states and possibly regulate the growth of the cancer cells. By incorporating CPP activity into the peptide sequence, the need for additional cellular delivery methods decreases. It is possible to design PPI inhibitors/activators from these protein sequences by fusing the CPPs to the peptides derived from other regions of the protein to form multifunctional peptides.

## Conclusion

In this work, we aimed to find new CPPs from nuclear proteins by using different prediction programs followed by the synthesis and the experimental testing of internalization. We assessed if the peptides have any effect on the cellular processes leading to the decrease of their proliferation. From each protein sequence, we had several hundreds of potential CPP sequences predicted. We can define the regions from the proteins, from which new peptides with high probability of being CPPs could be derived. Out of these, we chose one candidate per protein. The peptides were able to enter the cells and could be used further as delivery vectors. In this work, we found three peptides, AKIP1_27-37_, CASC3_251-264_, and AHRR_8-24_, which can enter the cells and could be further used to design new multifunctional bioactive peptides with internalization capability.

## Data Availability

The original contributions presented in the study are included in the article/Supplementary Material; further inquiries can be directed to the corresponding author.
