# Supplementary material for: Cell-Penetrating Peptides Predicted From CASC3, AKIP1, and AHRR Proteins
Source: Front Pharmacol. 2021 Aug 24;12:716226. doi: 10.3389/fphar.2021.716226 (PMC8421526; doi:10.3389/fphar.2021.716226)
Supplement: Supplementary file 1 [file DataSheet1.PDF]

Supplementary material

Table S1. Protein sequences used for CPP prediction, their length and number of predicted CPP sequences. Prediction described in (Hällbrink M, et al 2005 and Hansen M, et al 2008). Marking (Yellow) of the most probable CPP region in the protein.

<table>
<tr><td>Protein ID and sequence</td><td>Length</td><td>Predicted CPP sequences</td><td>Out of these NLS and/or sled motif containing</td></tr>
<tr><td colspan="4">>sp|Q9NQ31|AKIP1_HUMAN A-kinase-interacting protein 1 OS=Homo sapiens OX=9606 GN=AKIP1 PE=1 SV=2</td></tr>
<tr><td>MDNCLAAAALNGVDRRSLQRSARLALEVLERAKRRAVDWHALERPKGCMGVLAREAPHLEKQPAAGP
QRVLPGEREERPPTLSASFRTMAEFMDYTSSQCGKYYSSVPEEGGATHVYRYHRGESKLHMCLDIGNGQ
RKDRKKTSLGPGGSYQISEHAPEASQPAENISKDLYIEVYPGTYSVTVGSNDLTKKTHVVAVDSGQSVDL
VFPV</td><td>210 aa</td><td>322 (466)</td><td>49</td></tr>
<tr><td colspan="4">>sp|O15234|CASC3_HUMAN Protein CASC3 OS=Homo sapiens OX=9606 GN=CASC3 PE=1 SV=2</td></tr>
<tr><td>MADRRRQRASQDTEDEESGASGSDSGGSPLRGGGSCSGSAGGGGSGSLPSQRGGRTGALHLRRVESGGA
KSAEESECESEDGIEGDAVLSDYESAEDSEGEEGEYSEEENSKVELKSEANDAVNSSTKEEKGEEKPDTKS
TVTGERQSGDGQESTEPVENKVGKKGPKHLDDDEDRKNPAYIPRKGLFFEHDLRGQTQEEEVRPKGRQR
KLWKDEGRWEHDKFREDEQAPKSRQELIALYGYDIRSAHNPDDIKPRRIRKPRYGSPPQRDPNWNGERL
NKSHRHQGLGGTLPPRTFINRNAAGTGRMSAPRNYSRSGGFKEGRAGFRPVEAGGQHGGRSGETVKHEI
SYRSRRLEQTSVRDPSPEADAPVLGSPEKEEAASEPPAAAPDAAPPPPDRPIEKKSYSRARRTRTKVGDAV
KLAEEVPPPPEGLIPAPPVPETTPTPPTKTGTWEAPVDSSTSGLEQDVAQLNIAEQNWSPGQPSFLQPRELR
GMPNHIHMGAGPPPQFNRMEEMGVQGGRAKRYSSQRQRPVPEPPAPPVHISIMEGHYYDPLQFQGPIYT
HGDSPAPLPPQGMLVQPGMNLPHPGLHPHQTPAPLPNPGLYPPPVSMSPGQPPPQQLLAPTYFSAPGVMN
FGNPSYPYAPGALPPPPPPHLYPNTQAPSQVYGGVTYYNPAQQQVQPKPSPPRRTPQPVTIKPPPPEVVSR
GSS</td><td>703 aa</td><td>86 (521)</td><td>28</td></tr>
<tr><td colspan="4">>sp|Q96S94|CCNL2_HUMAN Cyclin-L2 OS=Homo sapiens OX=9606 GN=CCNL2 PE=1 SV=1</td></tr>
<tr><td>MAAAAAAAGAAGSAAPAAAAGAPGSGGAPSGSQGVLIGDRLYSGVLITLENCLLPDDKLRFTPSMSSGL
DTDTETDLRVVGCELIQAAGILLRLPQVAMATGQVLFQRFFYTKSFVKHSMEHVSMACVHLASKIEEAPR
RIRDVINVFHRLRQLRDKKKPVPLLLDQDYVNLKNQIIKAERRVLKELGFCVHVKHPHKIIVMYLQVLEC
ERNQHLVQTSWNYMNDSLRTDVFVRFQPESIACACIYLAARTLEIPLPNRPHWFLLFGATEEEIQEICLKIL
QLYARKKVDLTHLEGEVEKRKHAIEEAKAQARGLLPGGTQVLDGTSGFSPAPKLVESPKEGKGSKPSPLS
VKNTKRRLEGAKKAKADSPVNGLPKGRESRSRSRSREQSYSRSPSRSASPKRRKSDGSTSGGSKSQSRSR</td><td>520 aa</td><td>319 (523)</td><td>154</td></tr>
</table>

<table>
<tr><td>SRSDSPPRQAPRSAPYKGSEIRGSRKSKDCKYPQKPHKSRSRSSSRSRSRSRERADNPGKYKKKSHYYRD
QRRERSRSYERTGRRYERDHPGHSRHRR</td><td></td><td></td><td></td></tr>
<tr><td colspan="4">>sp|Q9UNL4|ING4_HUMAN Inhibitor of growth protein 4 OS=Homo sapiens OX=9606 GN=ING4 PE=1 SV=1</td></tr>
<tr><td>MAAGMYLEHYLDSIENLPFELQRNFQLMRDLDQRTEDLKAEIDKLATEYMSSARSLSSEEKLALLKQIQE
AYGKCKEFGDDKVQLAMQTYEMVDKHIRRLDTDLARFEADLKEKQIESSDYDSSSSKGKKKGRTQKEK
KAARARSKGKNSDEEAPKTAQKKLKLVRTSPEYGMPSVTFGSVHPSDVLDMPVDPNEPTYCLCHQVSY
GEMIGCDNPDCSIEWFHFACVGLTTKPRGKWFCPRCSQERKKK</td><td>249 aa</td><td>152 (179)</td><td>22</td></tr>
<tr><td colspan="4">>sp|P53355|DAPK1_HUMAN Death-associated protein kinase 1 OS=Homo sapiens OX=9606 GN=DAPK1 PE=1 SV=6</td></tr>
<tr><td>MTVFRQENVDDYYDTGEELGSGQFAVVKKCREKSTGLQYAAKFIKKRRTKSSRRGVSREDIEREVSILKE
IQHPNVITLHEVYENKTDVILILELVAGGELFDFLAEKESLTEEEATEFLKQILNGVYYLHSLQIAHFDLKPE
NIMLLDRNVPKPRIKIIDFGLAHKIDFGNEFKNIFGTPEFVAPEIVNYEPLGLEADMWSIGVITYILLSGASPF
LGDTKQETLANVSAVNYEFEDEYFSNTSALAKDFIRRLLVKDPKKRMTIQDSLQHPWIKPKDTQQALSRK
ASAVNMEKFKKFAARKKWKQSVRLISLCQRLSRSFLSRSNMSVARSDDTLDEEDSFVMKAIIHAINDDNV
PGLQHLLGSLSNYDVNQPNKHGTPPLLIAAGCGNIQILQLLIKRGSRIDVQDKGGSNAVYWAARHGHVDT
LKFLSENKCPLDVKDKSGEMALHVAARYGHADVAQLLCSFGSNPNIQDKEEETPLHCAAWHGYYSVAK
ALCEAGCNVNIKNREGETPLLTASARGYHDIVECLAEHGADLNACDKDGHIALHLAVRRCQMEVIKTLL
SQGCFVDYQDRHGNTPLHVACKDGNMPIVVALCEANCNLDISNKYGRTPLHLAANNGILDVVRYLCLM
GASVEALTTDGKTAEDLARSEQHEHVAGLLARLRKDTHRGLFIQQLRPTQNLQPRIKLKLFGHSGSGKTT
LVESLKCGLLRSFFRRRRPRLSSTNSSRFPPSPLASKPTVSVSINNLYPGCENVSVRSRSMMFEPGLTKGML
EVFVAPTHHPHCSADDQSTKAIDIQNAYLNGVGDFSVWEFSGNPVYFCCYDYFAANDPTSIHVVVFSLEE
PYEIQLNQVIFWLSFLKSLVPVEEPIAFGGKLKNPLQVVLVATHADIMNVPRPAGGEFGYDKDTSLLKEIR
NRFGNDLHISNKLFVLDAGASGSKDMKVLRNHLQEIRSQIVSVCPPMTHLCEKIISTLPSWRKLNGPNQL
MSLQQFVYDVQDQLNPLASEEDLRRIAQQLHSTGEINIMQSETVQDVLLLDPRWLCTNVLGKLLSVETPR
ALHHYRGRYTVEDIQRLVPDSDVEELLQILDAMDICARDLSSGTMVDVPALIKTDNLHRSWADEEDEVM
VYGGVRIVPVEHLTPFPCGIFHKVQVNLCRWIHQQSTEGDADIRLWVNGCKLANRGAELLVLLVNHGQG
IEVQVRGLETEKIKCCLLLDSVCSTIENVMATTLPGLLTVKHYLSPQQLREHHEPVMIYQPRDFFRAQTLK
ETSLTNTMGGYKESFSSIMCFGCHDVYSQASLGMDIHASDLNLLTRRKLSRLLDPPDPLGKDWCLLAMN
LGLPDLVAKYNTSNGAPKDFLPSPLHALLREWTTYPESTVGTLMSKLRELGRRDAADFLLKASSVFKINL
DGNGQEAYASSCNSGTSYNSISSVVSR</td><td>1430 aa</td><td>476 (1243)</td><td>21</td></tr>
<tr><td colspan="4">>sp|Q9NPF5|DMAP1_HUMAN DNA methyltransferase 1-associated protein 1 OS=Homo sapiens OX=9606 GN=DMAP1 PE=1 SV=1</td></tr>
<tr><td>MATGADVRDILELGGPEGDAASGTISKKDIINPDKKKSKKSSETLTFKRPEGMHREVYALLYSDKKDAPP
LLPSDTGQGYRTVKAKLGSKKVRPWKWMPFTNPARKDGAMFFHWRRAAEEGKDYPFARFNKTVQVPV
YSEQEYQLYLHDDAWTKAETDHLFDLSRRFDLRFVVIHDRYDHQQFKKRSVEDLKERYYHICAKLANV
RAVPGTDLKIPVFDAGHERRRKEQLERLYNRTPEQVAEEEYLLQELRKIEARKKEREKRSQDLQKLITAA
DTTAEQRRTERKAPKKKLPQKKEAEKPAVPETAGIKFPDFKSAGVTLRSQRMKLPSSVGQKKIKALEQM</td><td>467 aa</td><td>348 (821)</td><td>287</td></tr>
</table>

LLELGVELSPTPTEELVHMFNELRSDLVLLYELKQACANCEYELQMLRHRHEALARAGVLGGPATPASG PGPASAEPAVTEPGLGPDPKDTIIDVVGAPLTPNSRKRRESASSSSSVKKAKKP			
>sp|Q9NZM5|NOP53_HUMAN Ribosome biogenesis protein NOP53 OS=Homo sapiens OX=9606 GN=NOP53 PE=1 SV=2			
MAAGGSGVGGKRSSKSDADSGFLGLRPTSVDPALRRRRRGPRNKKRGWRRLAQEPLGLEVDQFLEDVR LQERTSGGLLSEAPNEKLFFVDTGSKEKGLTKKRTKVQKKSLLLKKPLRVDLILENTSKVPAPKDVLAHQ VPNAKKLRRKEQLWEKLAKQGELPREVRRAQARLLNPSATRAKPGPQDTVERPFYDLWASDNPLDRPL VGQDEFFLEQTKKKGVKRPARLHTKPSQAPAVEVAPAGASYNPSFEDHQTLLSAAHEVELQRQKEAEKL ERQLALPATEQAATQESTFQELCEGLLEESDGEGEPGQGEGPEAGDAEVCPTPARLATTEKKTEQQRRRE KAVHRLRVQQAALRAARLRHQELFRLRGIKAQVALRLAELARRQRRRQARREAEADKPRRLGRLKYQA PDIDVQLSSELTDSLRTLKPEGNILRDRFKSFQRRNMIEPRERAKFKRKYKVKLVEKRAFREIQL	478 aa	1178 (2528)	17
>sp|A9YTQ3|AHRR_HUMAN Aryl hydrocarbon receptor repressor OS=Homo sapiens OX=9606 GN=AHRR PE=1 SV=3			
MPRTMIPPGECTYAGRKRRRPLQKQRPAVGAEKSNPSKRHRDRLNAELDHLASLLPFPPDIISKLDKLSVL RLSVSYLRVKSFFQVVQEQSSRQPAAGAPSPGDSCPLAGSAVLEGRLLLESLNGFALVVSAEGTIFYASAT IVDYLGFHQTDVMHQNIYDYIHVDDRQDFCRQLHWAMDPPQVVFGQPPPLETGDDAILGRLLRAQEWG TGTPTEYSAFLTRCFICRVRCLLDSTSGFLTMQFQGKLKFLFGQKKKAPSGAMLPPRLSLFCIAAPVLLPSA AEMKMRSALLRAKPRADTAATADAKVKATTSLCESELHGKPNYSAGRSSRESGVLVLREQTDAGRWAQ VPARAPCLCLRGGPDLVLDPKGGSGDREEEQHRMLSRASGVTGRRETPGPTKPLPWTAGKHSEDGARPR LQPSKNDPPSLRPMPRGSCLPCPCVQGTFRNSPISHPPSPSPSAYSSRTSRPMRDVGEDQVHPPLCHFPQRS LQHQLPQPGAQRFATRGYPMEDMKLQGVPMPPGDLCGPTLLLDVSIKMEKDSGCEGAADGCVPSQVWL GASDRSHPATFPTRMHLKTEPDSRQQVYISHLGHGVRGAQPHGRATAGRSRELTPFHPAHCACLEPTDGL PQSEPPHQLCARGRGEQSCTCRAAEAAPVVKREPLDSPQWATHSQGMVPGMLPKSALATLVPPQASGCT FLP	701 aa	197 (381)	4
>sp|Q13620|CUL4B_HUMAN Cullin-4B OS=Homo sapiens OX=9606 GN=CUL4B PE=1 SV=4			
MMSQSSGSGDGNDDEATTSKDGGFSSPSPSAAAAAQEVRSATDGNTSTTPPTSAKKRKLNSSSSSSSNSS NEREDFDSTSSSSSTPPLQPRDSASPSTSSFCLGVSVAASSHVPIQKKLRFEDTLEFVGFDAKMAEESSSSS SSSPTAATSQQQQLKNKSILISSVASVHHANGLAKSSTTVSSFANSKPGSAKKLVIKNFKDKPKLPENYTD ETWQKLKEAVEAIQNSTSIKYNLEELYQAVENLCSYKISANLYKQLRQICEDHIKAQIHQFREDSLDSVLF LKKIDRCWQNHCRQMIMIRSIFLFLDRTYVLQNSMLPSIWDMGLELFRAHIISDQKVQNKTIDGILLLIERE RNGEAIDRSLLRSLLSMLSDLQIYQDSFEQRFLEETNRLYAAEGQKLMQEREVPEYLHHVNKRLEEEADR LITYLDQTTQKSLIATVEKQLLGEHLTAILQKGLNNLLDENRIQDLSLLYQLFSRVRGGVQVLLQQWIEYI KAFGSTIVINPEKDKTMVQELLDFKDKVDHIIDICFLKNEKFINAMKEAFETFINKRPNKPAELIAKYVDSK LRAGNKEATDEELEKMLDKIMIIFRFIYGKDVFEAFYKKDLAKRLLVGKSASVDAEKSMLSKLKHECGA AFTSKLEGMFKDMELSKDIMIQFKQYMQNQNVPGNIELTVNILTMGYWPTYVPMEVHLPPEMVKLQEIF	913 aa	309 (452)	44

KTFYLGKHSGRKLQWQSTLGHCVLKAEFKEGKKELQVSLFQTLVLLMFNEGEEFSLEEIKQATGIEDGEL RRTLQSLACGKARVLAKNPKGKDIEDGDKFICNDDFKHKLFRIKINQIQMKETVEEQASTTERVFQDRQY QIDAAIVRIMKMRKTLSHNLLVSEVYNQLKFPVKPADLKKRIESLIDRDYMERDKENPNQYNYIA			
>sp|Q969P5|FBX32_HUMAN F-box only protein 32 OS=Homo sapiens OX=9606 GN=FBXO32 PE=1 SV=1			
MPFLGQDWRSPGQNWVKTADGWKRFLDEKSGSFVSDLSSYCNKEVYNKENLFNSLNYDVAAKKRKKD MLNSKTKTQYFHQEKWIYVHKGSTKERHGYCTLGEAFNRLDFSTAILDSRRFNYVVRLLELIAKSQLTSL SGIAQKNFMNILEKVVLKVLEDQQNIRLIRELLQTLYTSLCTLVQRVGKSVLVGNINMWVYRMETILHW QQQLNNIQITRPAFKGLTFTDLPLCLQLNIMQRLSDGRDLVSLGQAAPDLHVLSEDRLLWKKLCQYHFSE RQIRKRLILSDKGQLDWKKMYFKLVRCYPRKEQYGDTLQLCKHCHILSWKGTDHPCTANNPESCSVSLS PQDFINLFKF	355 aa	47 (300)	25

Table S2. Calculated parameters for predicted peptide sequences. Based on calculations on CellPPD.

Origin	Peptide sequence derived from protein	Peptide name	Charge	M$_w$	Hydrophobicity/ Hydrophilicity	pI	Hydrophilicity
HIV TAT	GRKKRRQRRRPPQ	Tat$_{48-60}$	+8	1719.21	-1.09/ 1.88	12.71	1.88
A-kinase-interacting protein 1	VLERAKRRAV	AKIP1$_{27-37}$	+3	1997.58	-0.49/ 0.92	11.72	0.92
CASC3	PDDIKPRRIRKPRY	CASC3$_{251-264}$	+4	1810.32	-0.67/ 1.29	10.94	1.29
Cyclin L2	NTKRRLEGAKKA	CCNL2$_{354-365}$	+4	1371.77	-0.59/ 1.25	11.10	1.25
Death-associated protein kinase 1	AAKFIKKRRTKSS	DAPK1$_{40-52}$	+6	1521.01	-0.52/ 0.99	12.04	0.99
Inhibitor of growth protein 4	TQKEKKAARARSK	ING4$_{134-145}$	+5	1501.92	-0.69/ 1.51	11.17	1.51
DNA methyltransferase 1-associated protein 1	RKRRESASSSSSVKKAKKP	DMAP1$_{459-467}$	+7	2117.68	-0.63/ 1.38	11.76	1.38
Predicted model CPP	KRKGRLRSKGKK	VP8	+8	1441.96	-0.85/ 1.88	12.32	1.88
Ribosome biogenesis protein NOP53	AEADKPRRLGRLK	NOP53$_{397-410}$	+3	1509.95	-0.55/ 1.26	10.91	1.26
Aryl hydrocarbon receptor repressor	GECTYAGRKRRRPLQK	AHRR$_{8-24}$	+5	1919.46	-0.60/ 0.95	10.95	0.95
Cullin-4B	TPPTSAKKRKL	CUL4B$_{48-59}$	+4	1226.63	-0.46/ 0.84	11.27	0.84
F-box only protein 32	VAAKKRKKDML	FBXO32$_{59-69}$	+4	1287.78	-0.46/ 1.13	10.47	1.13

Table S4. 2-Way ANOVA for A) cell lysate, B) flow cytometry and C) MTS assay results comparing the differences for the same peptide between cell lines U87, HeLa and PC3 for a and b. D) Cell lysate results compared to control untreated and E) flow cytometry results compared to untreated. The results comparing treatment group to untreated cells c. P > 0.05 – ns; p < 0.05 - *; p < 0.01 - **; p < 0.001 - ***.

**A**	HeLa vs PC3	HeLa vs U87	PC3 vs U87
Tat	ns	ns	ns
AKIP1	ns	ns	ns
CASC3	ns	ns	ns
CCNL2	ns	ns	ns
DAPK1	ns	ns	ns
ING4	ns	ns	ns
DMAP1	ns	ns	ns
VP8	ns	ns	ns
NOP53	ns	ns	ns
AHRR	ns	ns	***
CUL4B	ns	ns	ns
FBXO32	ns	ns	ns
Untreated	ns	ns	ns

**B**	HeLa vs PC3	HeLa vs U87	PC3 vs U87
Tat	***	ns	***
AKIP1	***	ns	***
CASC3	**	ns	***
CCNL2	ns	ns	ns
DAPK1	ns	ns	ns
ING4	ns	**	ns
DMAP1	ns	ns	ns
VP8	**	ns	**
NOP53	ns	ns	ns
AHRR	*	ns	**
CUL4B	ns	ns	ns
FBXO32	ns	ns	ns
Untreated	ns	ns	ns

**C**	Peptide added (μM)				
Treatment group in Figure 3 24 h, compared to untreated	0	0.5	2	8	32
Tat	ns	ns	ns	ns	*
AKIP1	ns	ns	*	*	*
CASC3	ns	**	**	***	***
AHRR	ns	ns	*	*	**
Treatment group in Figure 3 48 h, compared to untreated	0	0.5	2	8	32
Tat	ns	ns	ns	*	***
AKIP1	ns	ns	*	*	**
CASC3	ns	*	*	*	***

AHRR	ns	**	***	***	***

D				
Treatment group in Figure 1A 2 h after addition of peptides	HeLa	PC3	U87	Al cell lines considered
Tat	***	***	***	***
AKIP1	***	***	***	***
CASC3	***	**	*	***
CCNL2	ns	ns	ns	ns
DAPK1	ns	ns	ns	ns
ING4	ns	ns	ns	ns
DMAP1	ns	ns	ns	*
VP8	ns	ns	ns	ns
NOP53	ns	ns	ns	ns
AHRR	**	***	ns	***
CUL4B	ns	ns	ns	ns
FBXO32	ns	ns	ns	ns

E				
Treatment group in Figure 1B 2 h after addition of peptides	HeLa	PC3	U87	All cell lines considered
Tat	***	***	***	***
AKIP1	***	***	***	***
CASC3	***	***	***	***
CCNL2	ns	ns	ns	ns
DAPK1	ns	***	***	***
ING4	ns	ns	*	ns
DMAP1	*	***	***	***
VP8	***	***	***	***
NOP53	ns	***	*	**
AHRR	***	***	***	***
CUL4B	***	***	***	***
FBXO32	ns	ns	ns	ns

Figure S1. Distribution of CPPs based on their origin (A) and distribution of CPPs entries based on the cargoes delivered (B). Data fron CPPSite 2.0 available (Agrawal P, et al, 2016)

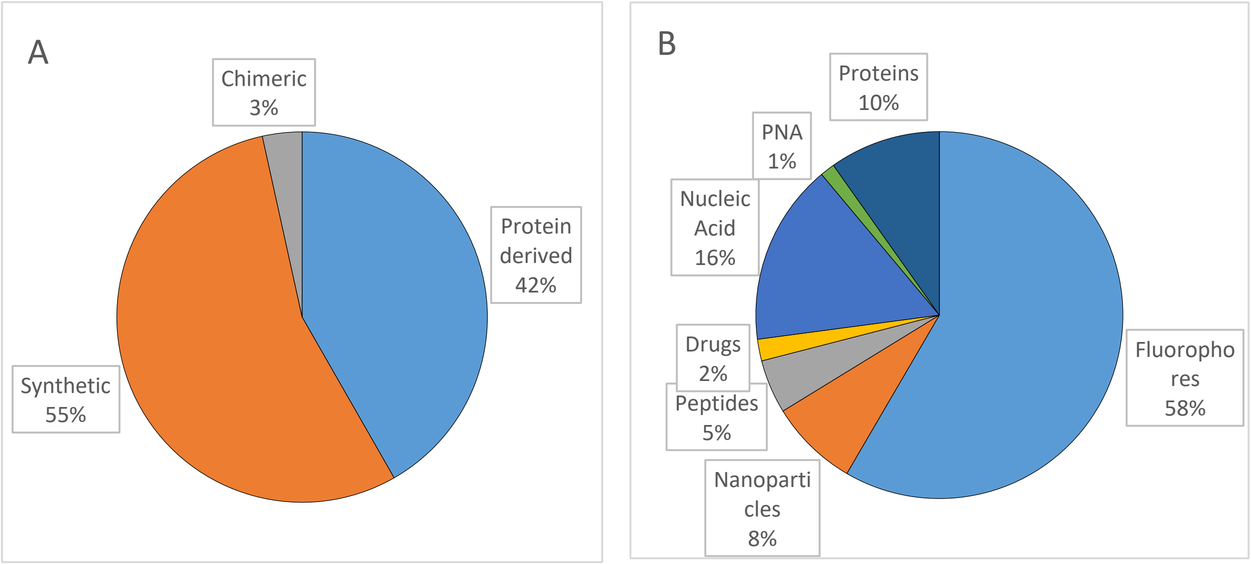

Figure S2. Internalisation of fluorescently labelled peptides. A) Corresponding flow cytometry analysis data to Figure 1 B in HeLa cells. After 2 h treatment with 10 µM peptide cells were washed, detached from the plate and outside fluorescence was quenched by addition of trypan blue. B) Internalisation of fluorescently labelled CASC3$_{251-264}$ and CPP Tat peptides (green) into HeLa cells. Cells were treated with 20 µM peptide solutions 4 h prior microscopy. Nuclei (blue) were stained with Hoechst. Images taken at 20 x magnification from live cells. C) Tat peptide treated control group and untreated group images shown in Figure 2, enlarged.

A)

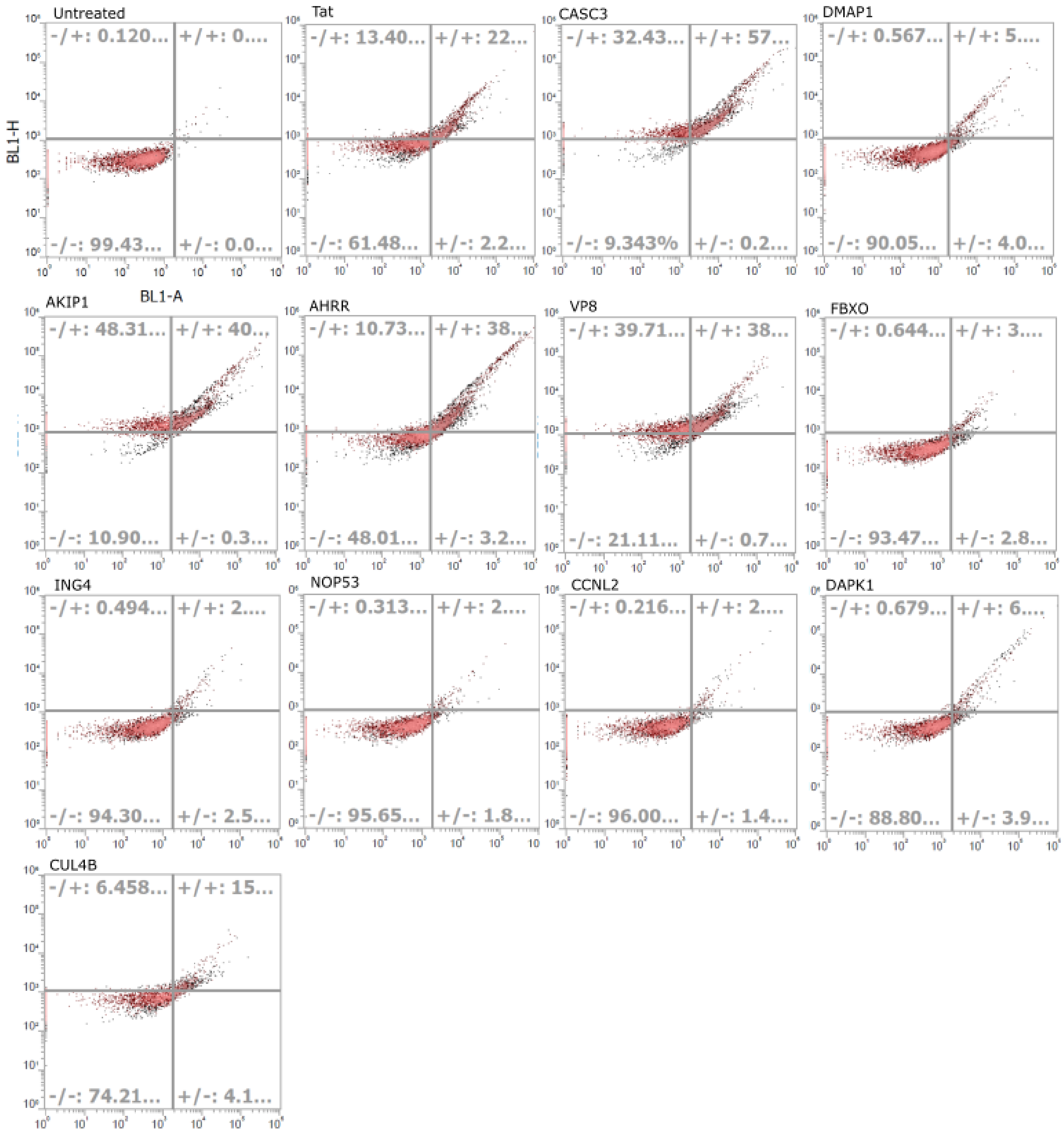

B)

Untreated Tat CASC3

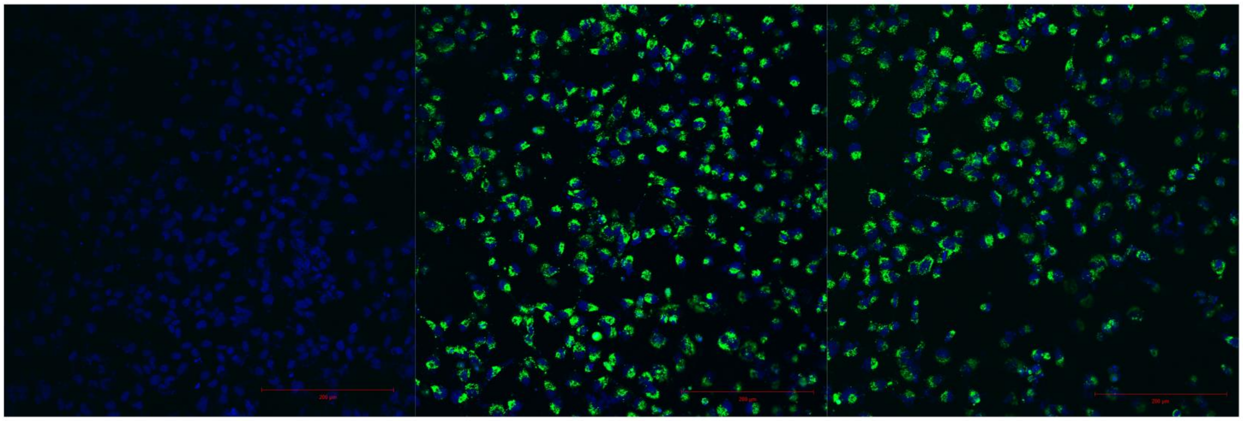

C)

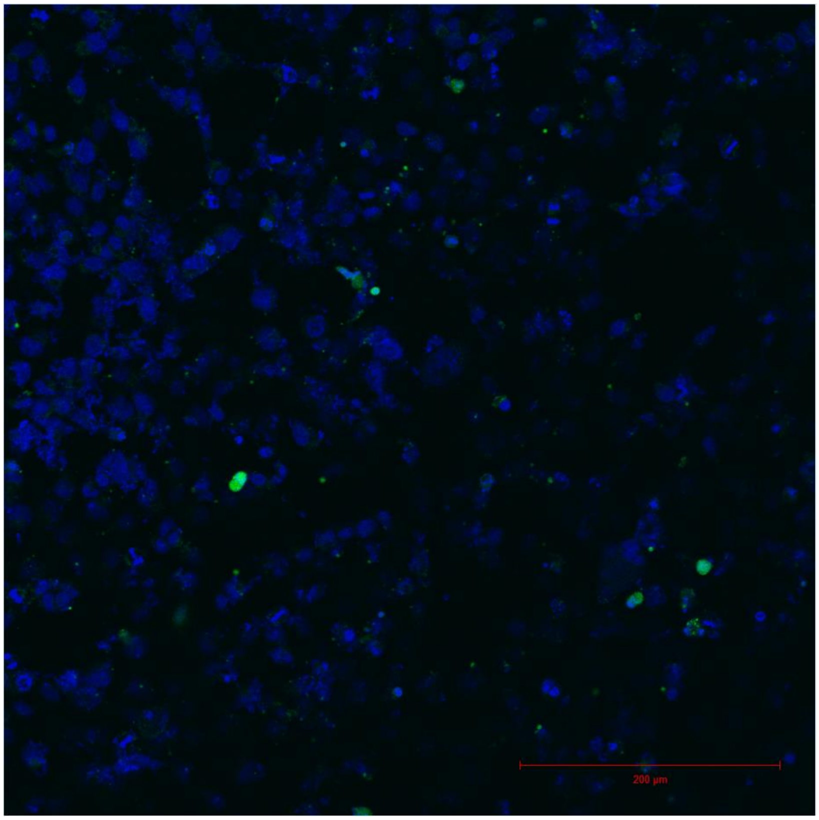

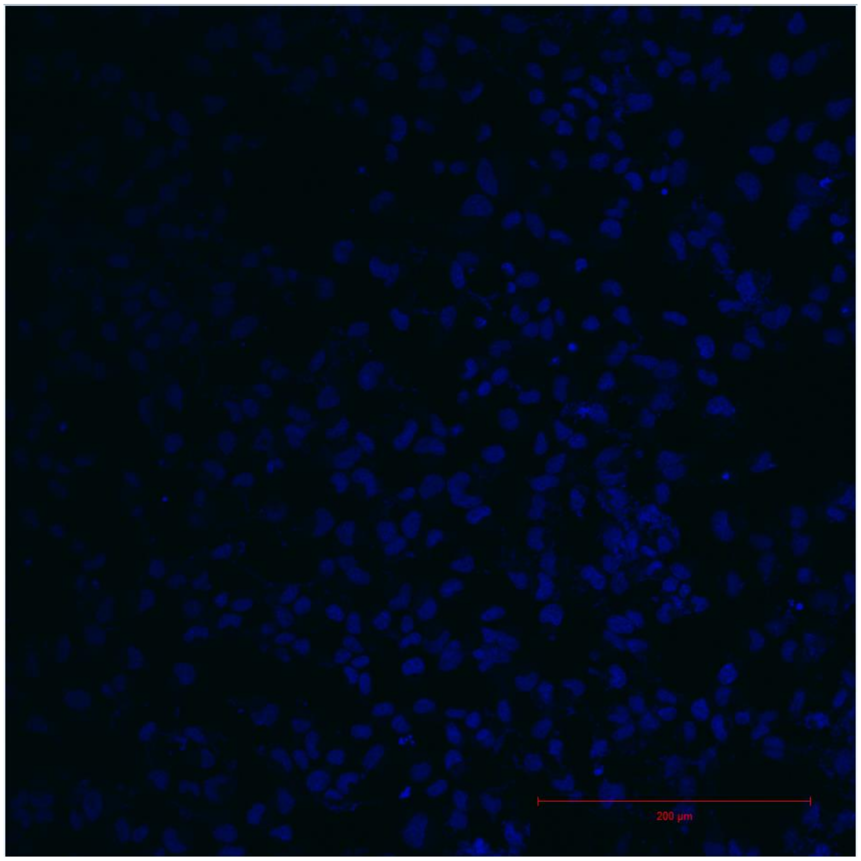

Figure S3. Total DNA from U87 cell lysate measured 48 h after addition of peptide solutions at given concentrations. For DNA quantification PicoGreen DNA binding fluorescent dye was used. Results are normalised to untreated cells.

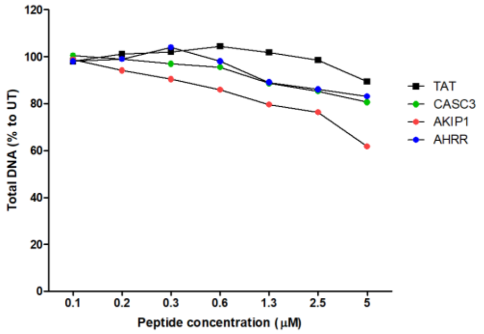

Figure S4. MTS assay on CHO. Results shown as an average of three independent wells. Measured 24 h and 48 h after addition of peptides. MTS reagent was added 4 h prior measurement.

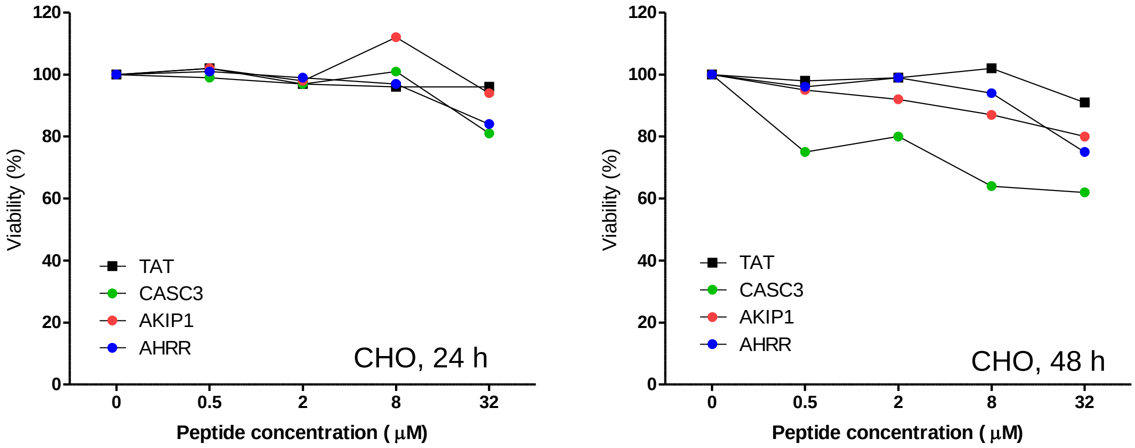

120
100
80
60
40
20
0
Viability (%)
TAT
CASC3
AKIP1
AHRR
CHO, 24 h
0
0.5
2
8
32
Peptide concentration (μM)
120
100
80
60
40
20
0
Viability (%)
TAT
CASC3
AKIP1
AHRR
CHO, 48 h
0
0.5
2
8
32
Peptide concentration (μM)
